# Retrospective Analysis for Genetic Improvement of Hip Joints of Cohort Labrador Retrievers in the United States: 1970–2007

**DOI:** 10.1371/journal.pone.0009410

**Published:** 2010-02-24

**Authors:** Yali Hou, Yachun Wang, George Lust, Lan Zhu, Zhiwu Zhang, Rory J. Todhunter

**Affiliations:** 1 College of Animal Science & Technology, China Agricultural University, Beijing, China; 2 Baker Institute for Animal Health, College of Veterinary Medicine, Cornell University, Ithaca, New York, United States of America; 3 Department of Statistics, Oklahoma State University, Stillwater, Oklahoma, United States of America; 4 Institute for Genomic Diversity, Cornell University, Ithaca, New York, United States of America; 5 Department of Clinical Sciences, College of Veterinary Medicine, Cornell University, Ithaca, New York, United States of America; Ohio State University Medical Center, United States of America

## Abstract

**Background:**

Canine Hip Dysplasia (CHD) is a common inherited disease that affects dog wellbeing and causes a heavy financial and emotional burden to dog owners and breeders due to secondary hip osteoarthritis. The Orthopedic Foundation for Animals (OFA) initiated a program in the 1960's to radiograph hip and elbow joints and release the OFA scores to the public for breeding dogs against CHD. Over last four decades, more than one million radiographic scores have been released.

**Methodology/Principal Findings:**

The pedigrees in the OFA database consisted of 258,851 Labrador retrievers, the major breed scored by the OFA (25% of total records). Of these, 154,352 dogs had an OFA hip score reported between 1970 and 2007. The rest of the dogs (104,499) were the ancestors of the 154,352 dogs to link the pedigree relationships. The OFA hip score is based on a 7-point scale with the best ranked as 1 (excellent) and the worst hip dysplasia as 7. A mixed linear model was used to estimate the effects of age, sex, and test year period and to predict the breeding value for each dog. Additive genetic and residual variances were estimated using the average information restricted maximum likelihood procedure. The analysis also provided an inbreeding coefficient for each dog. The hip scores averaged 1.93 (±SD = 0.59) and the heritability was 0.21. A steady genetic improvement has accrued over the four decades. The breeding values decreased (improved) linearly. By the end of 2005, the total genetic improvement was 0.1 units, which is equivalent to 17% of the total phenotypic standard deviation.

**Conclusion/Significance:**

A steady genetic improvement has been achieved through the selection based on the raw phenotype released by the OFA. As the heritability of the hip score was on the low end (0.21) of reported ranges, we propose that selection based on breeding values will result in more rapid genetic improvement than breeding based on phenotypic selection alone.

## Introduction

There are an estimated 60–70 million pet dogs in USA households. The selective crossing, and then line breeding, that generated pure dog breeds inadvertently caused fixation of recessive alleles that predispose to inherited disorders. Canine Hip Dysplasia (CHD) is a developmental disease that is expressed between 2 and 6 months of age for most medium and large dog breeds. It is a major veterinary medical problem with estimates of over 40% dysplastic dogs in some large breeds [1] and up to 75% as reported by the Orthopedic Foundation for Animals (OFA) (www.offa.org). Inherited developmental traits like hip and elbow dysplasia cause much of the osteoarthritis that plagues dogs as they age and could be prevented by better breeding practices. Treatment of a chronic disease like hip osteoarthritis, which results from hip dysplasia, has both a financial and emotional impact when it requires lifelong therapy or surgery. Pelvic radiographic examination of anesthetized or deeply sedated dogs in a ventro-dorsal position has been used in attempts to improve hip joint conformation and thus reduce the occurrence of hip dysplasia in dogs [2], [3], [4], [5], [6].

Canine hip dysplasia is a complex trait which can be characterized by at least four measurements. The OFA hip score [2] is the most popular measurement used in North America. The other three measurements are the Norberg angle (NA) [7], the distraction index (DI; PennHip™) [4] and the dorsolateral subluxation (DLS) score [6], [8]. Here we focus on a single method established by the Orthopedic Foundation for Animals (OFA) [2]. The OFA established a registry to certify dogs based on their hip conformation. Their scoring system includes the grades of excellent, good and fair for so-called unaffected dogs, a borderline grade, and grades of mild, moderate and severe for dogs with hip dysplasia (www.offa.org/hipgrade).

Selective breeding using normal dogs can reduce the frequency of hip dysplasia especially when selection of breeders is based on breeding values for hip dysplasia [9], [10]. In a previous report [11], analysis of OFA data suggested that for the period of 1970–1990 hip joint phenotypes improved in particular breeds. The percentage of dogs that were classified as excellent increased over this time. This phenotypic improvement has continued in their most recent analysis of OFA hip scores between 1989 and 2003 in the Labrador retriever, the Golden retriever, the German shepherd dog, the Bernese mountain dog, the rottweiler and other breeds combined [12]. The question that remains is whether the improvement underlying the phenotypic change has a genetic basis. Such a conclusion would offer support for the voluntary resource that the OFA provided because improvement in genetic hip quality should be sustainable.

Recently the genetic trends of the four measurements of CHD (OFA score, NA, DLS and DI) were reported for the Labrador retriever dogs in the breeding program of the Guiding Eyes for Blind in Yorktown Heights NY which formed part of a larger study [13]. Hip radiographic measurements (phenotypes) and pedigree information were jointly used to predict the genetic basis underlying each hip radiographic phenotype. Application of breeding values has previously been used for successful genetic improvement in livestock [14] and to reduce the incidence and severity of hip dysplasia in closed colonies of dogs [10]. In a recent report [13], the application of hip breeding values to the selection process of the Guiding Eyes for the Blind has resulted in genetic improvement in hip conformation. To extend this idea to the general population of pure breed registered dogs, we used hip phenotypes based on ventrodorsal pelvic radiographs of Labrador retriever dogs in the public OFA database (OFA scores) to evaluate whether the genetic improvement accrued over a period of almost four decades. Our hypothesis was that there was no improvement in the predicted breeding value for OFA scores over the four decades.

We analyzed the pedigree and hip joint phenotypes of Labrador retrievers in the OFA database for the period of 1970–2007 and predicted the hip score breeding value for each dog to verify if there was genetic improvement over this period. These breeding values and inbreeding coefficients are available to the public through a searchable webpage (www.vet.cornell.edu/research/bvhip) to enhance selection criteria for dog owners, breeders, and buyers of Labrador retrievers.

## Results

### Effect of Time Period

**Figure 1:**
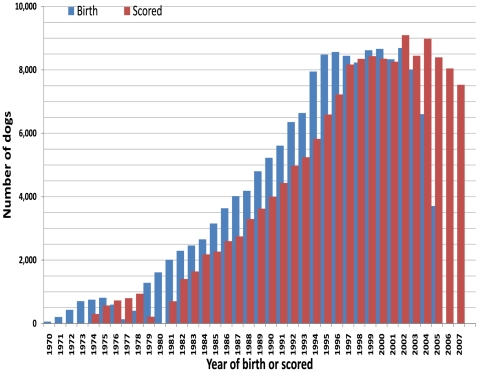
Distribution of dogs born and scored between 1970 and 2007. There were 154,352 Labrador retrievers scored by the Orthopedic Foundation for Animals (OFA) during this period. The number of dogs are indicated by the blue bars for the year that the dogs were born and by the red bars for the year that the dogs were scored. Both the minimum and the median age of scoring were two years old. The lag between the birth year and scoring year was over two years.

The number of dogs scored by OFA increased linearly each year since 1974. The increase reached a plateau in 1996 (Fig. 1). As most dogs were screened at 24 or more months of age, there was a two or more year of lag between the year of birth and the year in which the hips were scored. The OFA started reporting phenotype data in 1974. The founders of these dogs can be traced back to dogs born in 1970. The youngest dogs scored in 2007 were born in 2005. The dogs born in 2006 and 2007 have not been scored yet by the time this study made the final cut on scoring date. Because the number of dogs screened increased each year and the lag between year of birth and year of scoring was over two years, the number of dogs scored before 1997 was larger than the number of dogs that were born in the same year. The average number of dogs scored per year was 8,368 at the plateau with a standard deviation of 421.

During the last four decades, the OFA experienced four time periods when records were released under different policies. In period I (1970–1984), only the unaffected dogs were released as “Normal”. In the first half year of period II (1985), the release policy was as for in period I. In the second half year, the OFA started to release unaffected dogs with detailed categories. The policy was continued in the period III (1986–2000). In period IV (1986–2007), both affected and unaffected dogs were released with detailed categories (Table 1). No significant difference was found for the least square means across the four time periods (Table 2). This indicated that the scoring standard has been consistently followed over the past four decades. The OFA hip scores averaged 1.93 with a standard deviation of 0.59 among all the dogs investigated.

**Table 1:** Numerical coding and distribution of Orthopedic Foundation for Animals (OFA) scores*.

Category	Score	Time period				Total
		1970–1984	1985	1986–2000	2001–2007	
NORMAL	2	9,482	1,260			10,742
EXCELLENT	1		140	16,386	12,811	29,337
GOOD	2		740	57,019	39,501	97,260
FAIR	3		128	10,452	5,664	16,244
BOARDLINE	4				93	93
MILD	5				321	321
MODERATE	6				272	272
SEVERE	7				83	83
TOTAL		9,482	2,268	83,857	58,745	154,352

*The OFA scores were reported between 1970 and 2007. Four time periods were categorized according to the year the pelvic radiographs were taken and reported in the public OFA database based on record release policy.

**Table 2:** Statistics of Orthopedic Foundation for Animals (OFA) scores over the four time periods.

Period	N	Percent	Phenotypic mean	Phenotypic SD	LS Mean	LS Error
1970–1984	9,482	6.14	2.0000	0.0000	0.1178^ ns^	0.1251
1985	2,268	1.47	1.9947	0.3438	0.0931^ ns^	0.1144
1986–2000	83,857	54.33	1.9292	0.5613	0.1012^ ns^	0.0774
2001–2007	58,745	38.06	1.9219	0.6845	0.0000^ ns^	0.0000

ns – no significant difference at the level of 5%.

LS – least squares.

SD – standard deviation.

### The Contribution of Sires versus Dams

There were many less sires than dams for breeding dogs. Consequently, the average number of progeny per sire was 1.63 times of the number of progeny per dam. The variation of number of progeny per sire was much larger (6 fold) than the variation of number of progeny per dam (Table 3). The maximum number of progeny per sire was about 24 fold of the maximum number of progeny per dam (807 vs. 34). As the selection intensity among sires was higher than the intensity among dams, the selection of sires played a larger role in changing the genetic basis of CHD.

**Table 3:** The statistics of number of progeny per sire and per dam.

	N	mean	SD	SE	Min	Max
Sire	54,454	3.0252	10.7064	0.0459	1	807
Dam	88,698	1.8569	1.7400	0.0058	1	34

### Age and Sex Effect

The OFA score increased with age. The score measured on 37–60 month old dogs was significantly higher (P<0.05) than the score measured on 24 month old dogs (Table 4). No significant difference (P>0.05) in OFA hip scores was found between male and female dogs (Table 5).

**Table 4:** Statistics of Orthopedic Foundation for Animals (OFA) scores grouped by age.

Age (month)	N	Percent	Phenotypic mean	Phenotypic SD	LS Mean*	LS Error
24	38,766	25.12	1.9137	0.5951	−0.0221^b^	0.0091
25–29	55,100	35.70	1.9244	0.5856	−0.0137^ab^	0.0080
30–36	29,703	19.24	1.9549	0.5927	0.0096^ab^	0.0066
37–60	30,783	19.94	1.9453	0.6026	0.0000^a^	0.0000

*with different subscript indicating significantly different at 5% level.

LS – least squares.

SD – standard deviation.

**Table 5:** Statistics of Orthopedic Foundation for Animals (OFA) scores grouped by sex.

Sex	N	Percent	Phenotypic mean	Phenotypic SD	LS Mean	LS Error
Male	51,010	33.05	1.9317	0.5936	1.9944^ns^	0.0296
Female	103,342	66.95	1.9318	0.5926	1.9865^ns^	0.0295

ns – no significant difference at level of 5%.

LS – least squares.

SD – standard deviation.

### Variance Components and Heritability

The estimates of genetic and residual variance were 0.0729 and 0.2760, respectively. Consequently, OFA score in these Labrador retrievers had phenotypic variance of 0.3489 and a estimated heritability of 0.21 (Table 6).

**Table 6:** Estimates of variance components and heritability*.

	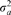	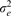	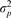	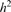
Estimate	0.0729	0.2760	0.3489	0.2086
Standard error	0.0020	0.0019	0.0014	0.0055

*The phenotypic variance (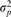) was decomposed into the additive genetic variance (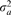) and the residual variance (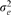). The heritability (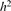) was calculated as the ratio of the additive genetic variance over the phenotypic variance.

### Phenotypic and Genetic Trends

**Figure 2:**
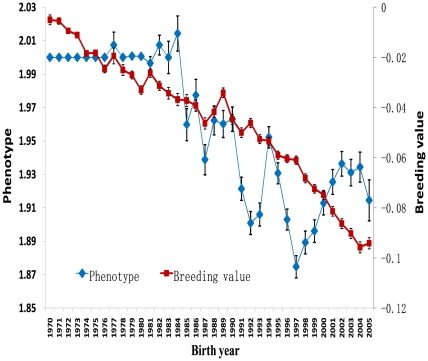
The phenotypic and genetic trends in last four decades. The trends are presented by the mean (± standard error) within each year for Orthopedic Foundation for Animals (OFA) score (the vertical axis on the left) and breeding value (the vertical axis on the right) for the 258,851 Labrador retrievers born between 1970 and 2005.

Both the phenotypic scores and the predicted breeding values improved steadily over the last four decades (Fig. 2). The phenotypic mean of OFA hip scores exhibited greater fluctuations than the breeding values. The breeding values improved linearly after integration of pedigree information and absorbing the influence of factors such as age when the radiographs were recorded. The improvement of breeding values was still found for dogs born between 1970 and 1983 even though the OFA scores in the public OFA database remained constant over that period. The selection based on the indicator of “normal” dogs still fostered genetic improvement. The total change in hip breeding values over the four decades was 0.1 units for the dogs born in 2005 compared to dogs born in 1970.

**Figure 3:**
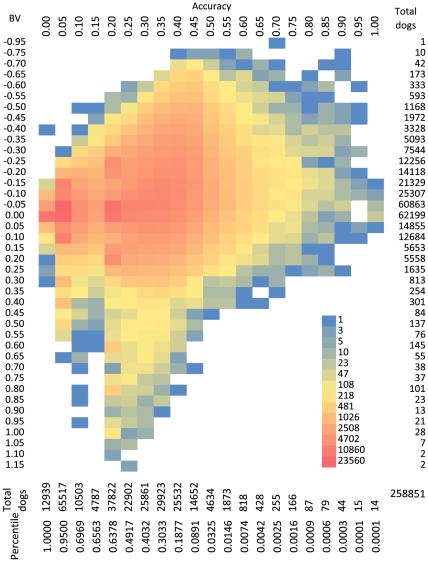
The joint and marginal distribution of breeding value and accuracy. Breeding value (BV) is displayed on the vertical axis and its accuracy is plotted on the horizontal axis for the 258,851 Labrador retrievers born between 1970 and 2005. The marginal distributions of BV and accuracy are indicated by both the total number of dogs and percentile at each category. The dogs on the upper right with lower BV (better hip) and higher accuracy are the most ideal for breeding against hip dysplasia.

A joint distribution of breeding values and their accuracies is displayed for the 258,851 Labrador retrievers in Fig. 3. A lower breeding value indicates better hip joint conformation as lower OFA scores indicate better hip conformation. The higher accuracy, the more precision for the corresponding breeding value because more dogs are used for its prediction. The dogs in the upper right corner of the plot in Fig. 3 have the most accurate and desirable hip joint conformation. The correlation between the breeding value and its accuracy was −0.32. This correlation (P<0.01) indicated that selection for hip quality had been employed upon the dogs in this database as suggested [12]. The dogs with lower breeding values (better hip conformation) in the upper right corner have been more intensively used to generate progeny. Consequently, these dogs achieved higher accuracy as more progeny were available upon which to make accurate estimates.

### Inbreeding

**Figure 4:**
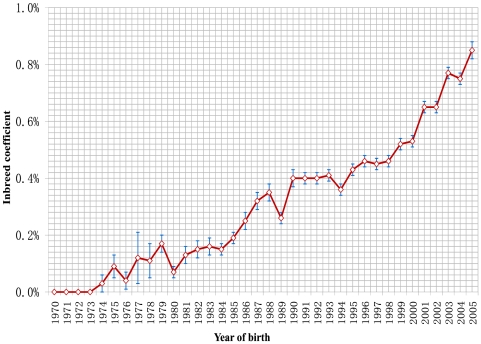
Trend of inbreeding coefficients. The trend is presented by the mean (± standard error) within each year for the 258,851 Labrador retrievers born between 1970 and 2005.

The average inbreeding coefficient was 0.85% for the dogs born in 2005. The founder dogs born in 1970–1973 were considered as unrelated and had inbreeding coefficients of zero. The average increment of inbreeding was 0.0283% per year over the past forty years (Fig. 4).

## Discussion

Our goal was to determine if improvement in hip phenotypes, as has been reported in the OFA database over the last four decades [11], [12], has been accompanied by improvement in a breed's genetic potential for nondysplastic hip conformation. We used as the prototype breed the Labrador retriever as one of the most popular breeds in North America. To do this, the pedigree and hip score information in the public OFA database were combined to derive hip breeding values. Our analysis clearly showed that irregular improvement in hip quality has been accompanied by steady improvement in the hip breeding values. The trend was negative as a better hip was classified with a lower OFA score in this analysis. According to Fig. 2, the total change in hip breeding value was 0.1 units which was 17% of the total phenotypic standard deviation (0.59). In the period of 2001–2007, when all seven categories of OFA scores were reported, the proportion of excellent hips was 22%. The cutoff for having an excellent hip score would be 0.77 standard deviations above the mean for the underlying breeding value. Under the assumption that the underlying breeding value had a normal distribution, the improvement of 17% of the standard deviation implied that the proportion of excellent hips in the public OFA data base would be increased to 27%. This meant that the proportion of excellent hips increased 24% during the last four decades. Thus, even for a database biased toward better hip conformation, measureable improvement could be achieved. The challenge is how to use this publicly available data most efficiently to maximize genetic improvement [15], [16], [17].

When dogs are bred as part of closed breeding colonies for a specific purpose, the decision of which dogs to breed is often made by staff geneticists. Breeding decisions then take many traits into account including those particular to the musculoskeletal system. When breeding decisions are made based on the breeding values for a particular trait like CHD, further reduction in unwanted traits can be achieved [10], [13], [18] compared to when decisions are made based on phenotype alone. Several investigators have advocated the application of hip breeding values to breeding decisions including Ginja et al. [19] most recently. However, individual dog breeders do not have access, for the most part, to a large diverse group of potential male and female dogs from which to breed. Most have a few female and male dogs and may search for a suitable mate. Individuals who plan to purchase a pure breed dog often do not have the time or the expertise to search public databases in order to find litters of puppies for which full pedigrees and hip radiographs are available. Based on our analysis, we are encouraged to deliver these hip breeding values and related inbreeding coefficients derived from the public OFA database to the breeders and dog owners to educate their choice of a mate or purchase of a dog, respectively. We reason that integration of this information into a user-friendly web based format will encourage more rapid improvement in Labrador retriever hip quality than the current *ad hoc* system (see Figure S1).

### Bias in the Data Base

The phenotypic data reported in the public OFA database was highly biased against CHD reporting. Only the dogs with excellent, good or fair hip scores were publicly reported in the period from 1970 to 2000. All the dogs were reported as “normal” until the second half year in 1985. Starting from 2001, the dysplastic dogs and dogs with borderline scores were reported (Table 1). In the OFA database (www.offa.org) accessed on August 31, 2009, 18% of Labrador retrievers were reported as dysplastic in the breed statistic summary. Yet when complete information of publicly identified dogs was accessed for the analysis reported here, less than 1% of dogs were dysplastic (OFA scores of mile, moderate and severe hip dysplasia). This indicated that a minor proportion of dysplastic dogs were reported in the publicly accessible part of the OFA database. Although this was not the best way of breeding against CHD, it implied that selection was applied as the reported dogs had more chances to breed. This is equivalent to the situation in livestock breeding. Individuals from undesirable families are usually omitted for phenotyping to reduce cost. Nevertheless, with the collected information, including the dog's pedigree, age, and sex, useful information for analysis was still extracted. Even though the OFA database is biased toward unaffected hips due to the optional nature of public submissions, taken together our results are encouraging. The statistical properties of breeding values resulting from the mixed model approach still hold for a population under selection. These properties are called best linear unbiased prediction (BLUP) [20]. Clearly, the more complete the pedigree and phenotype information included in the calculation of the breeding value, the more accurate the estimate.

### Popular Sire Effects

There was a strong sire effect in that as many as 807 puppies were bred by a single sire while the largest number of puppies produced by a single dam was 34. The variation of number of progeny per sire was six fold of the variation of the number of progeny per dam (Table 3). Using breeding values, several prospective sires or dams would be ranked by hip breeding values [13] and then selected according to many other traits deemed important to a particular breeder. Such a selection would inevitably lead to the “popular sire effect” because it is likely that the dogs with best hip quality would be used as sires more often. Actually, this trend already exists, even with the current selection on phenotypes. There are 104 dogs with accuracy>75% and breeding values <−0.25. Among these dogs, there were only two females, the rest (102) were males. The value of these dogs would increase accordingly. It is clear from the OFA data analyzed herein over the last four decades that many more offspring (24-fold more in this study) are produced per sire than per dam. Hence it is also important to provide inbreeding coefficients for each dog. Inbreeding coefficients did increase steadily over the analyzed years which is not unexpected if breeders continue to select the better hipped dogs for breeding. It is possible that even though breeders may carefully select a dog with which to breed that is a popular sire that inadvertent propagation of undesirable clandestine traits may ensue. As dog breeders make breeding decisions based on many factors, it is unlikely that deleterious inbreeding would occur in a large diverse breed like the Labrador retriever. In breeds with small effective population size however, attention to maintenance of genetic diversity would be important. Education in the best practice of the use of breeding values will be essential to maximize genetic diversity. Eventually, breeding values could be weighted for many qualities thought to be important to the breed. Some like orthopedic traits will be more easily quantified than others like behavior or hunting characteristics.

### Heritability

The heritability of OFA hip score in this study estimated at 0.21 is consistent with other reports that have used this, or a similar hip scoring system, but lower compared to other radiographic methods used to estimate heritability of CHD [21]. Selection on phenotypes is as accurate as the selection on breeding values only when heritability is close to one. Therefore, we reason that selection of dogs based on breeding values would improve hip quality more rapidly compared to selection based on raw OFA hip scores [9], [14], [22]. When radiographic methods that emphasize hip laxity in which the hips are imaged in a neutral position are used to estimate heritability in pedigrees, the estimates can be higher [13], [23]. Heritability estimates are also affected by the pedigree on which the calculations are made and missing information on the phenotype (as in this study) and the pedigree relatives. Fig. 1 shows that more dogs were born (i.e. had relatives in their pedigrees) than were reported as scored in the public OFA data until the late 1990's and this is consistent with comparisons to records of litters registered by the American Kennel Club [11] in which most dogs registered are not recorded with hip scores in the OFA database. Additionally, the expression of complex traits like CHD is also influenced by non genetic (environmental) factors like plane of nutrition, exercise and score variation among radiologists. Although the average concordance rate among the radiologists was reasonably good as reported by the OFA (74%), including the radiologist in the model would improve the accuracy. The estimate of heritability made on dogs in closed breeding colonies in which the environment is controlled is likely to be higher than the estimates made on the entire breed in a country.

### Selection Pressure

Selection pressure placed upon breeders based on traits with higher heritability will likely result in more rapid gain than selection pressure based on traits with lower heritability. Selection pressure based on the distraction index has been reported to markedly reduce the incidence of CHD in a closed population of Labrador retrievers and German shepherd dogs [10]. Our studies have shown the OFA score and the NA are highly genetically correlated while the DI and the DLS score are also highly genetically correlated but that the genetic correlations between these two groups of hip phenotypes are only moderate [13]. Therefore, selection pressure based on the OFA score will likely not result in as rapid gain as if selection pressure was also exerted based on either the DI or the DLS score which have higher heritability [23]. At present, there is no publicly accessible database for the DI or the DLS score. So for now, breeders and prospective purchasers must avail themselves of individual dog phenotype and related pedigree information to which they have access from breeders or be given access to publicly available hip scores and integrated pedigree information (breeding values) based on the public OFA database.

Modest improvement in hip score breeding values occurred through voluntary self-reporting and any voluntary improvements breeders have made in their breeding decisions. It is also possible that competition from PennHIP™, who also uses the extended hip radiograph to assess hip conformation, and reports a dog's DI relative to the breed as a whole, has improved hip conformation in American Labrador retrievers. Once breeders and prospective Labrador retriever owners are provided breeding values based on the OFA scores, we anticipate that more rapid gain in hip conformation will ensue as suggested by Kaneene et al. [12] who suggested more use of progeny testing. However, progeny testing for a complex trait requires the evaluation of many offspring (maybe as many as 15–20) to best estimate a dog's genetic potential. The provision of breeding values would obviate this step in selection of the best breeding dogs with reasonable accuracy for good hip conformation (see Figure S2). As some of these dogs have a DNA sample available for genotyping (www.caninehealthinfo.org), the application of these breeding values will also be used in studies leading to identification of contributing genes and to establish genomic prediction for susceptibility or resistance to CHD.

## Materials and Methods

### Dogs

The OFA database provides selection criteria for pet dog breeders and owners to lower the incidence of inherited disease. By the end of 2007, there were 1,411,127 entries reported in the public OFA database, 266,805 of which belonged to Labrador retriever dogs. There were 168,617 records on hip scores. The rest (98,188) were the records on other traits such as elbow dysplasia. Additionally, 14,265 hip records were removed for data quality control. The final data set used for the analysis contained 154,352 dogs each with one hip score record. The pedigrees recorded in the OFA data base provided an additional 104,499 ancestors (without hip scores) which were used to establish kinship among dogs with hip scores. The total number of dogs included in this analysis was 258,851. The trait records and pedigrees used in this retrospective study were available in the public domain and no live animals were used in the study.

Replicate dogs, dogs recorded with incorrect sex (such as female sire or male dam), or recorded as born earlier than a parent were also removed. These reasons lead to the deletion of 5,514 records. The most common age for scoring was 24 months (24%) and 71% of dogs were scored between 24 and 60 month. The rest (5%) of the dogs (8,751) scored before 24 months or after 60 months of age were removed from the analysis as part of data quality control.

As most of the dogs were scored at 2 years old, a lag of two year existed from the last date on which a dog was born (end of 2005) to when it was scored at the end of 2007. Therefore, the latest records used in the analysis dated from the end of 2007.

### Hip Phenotypes

Hip radiographs were taken by a veterinarian with the dog in the hip-extended, supine position (www.offa.org/hipproc) and were mailed to the OFA by the owner. The radiographs were independently evaluated by 3 randomly selected board-certified radiologists from a pool of 20–25, with concordance rates averaging 74% over 1.8 million radiographs, according to the OFA. Dogs were scored into seven categories: excellent, good, and fair hip conformation, borderline, then mild, moderate and severe hip dysplasia and each score was given a numerical value from 1 (excellent hip conformation) to 7 (severe hip dysplasia). The first three categories (excellent, good and fair) are considered by many as “normal” dogs. The last three categories (mild, moderate and severe) are considered “dysplastic” dogs.

From 1970 to mid 1985, only records of the normal dogs were released as “normal”. From mid 1985 to 2000, the “normal” dogs were released with their hip score categories (excellent, good and fair). From 2001, all seven score categories were released. Based on the record release policy, four time periods were grouped correspondingly, with year of 1985 as a separate group (Table 1). A numerical value of 2 was assigned to the combined category of “normal” for the only category reported in the first time period. Four test year periods were formed based on the record releasing policy of OFA: 1770–1984, 1985, 1986–2000 and 2000–2007.

The earliest ancestors were born in 1970. There were no further pedigrees that could be traced back further for the ancestors born between 1970 and 1973. These ancestors were therefore considered as founders with inbreeding coefficient of zero.

### Statistical Model

A single trait mixed linear model was employed to estimate the variance components (additive genetic variance and residual variance) and to predict breeding values for each dog in the pedigree. The model can be illustrated in matrix notation:
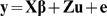
where **y** is the vector of observed phenotypes (OFA hip scores); **β** is the vector of fixed effects including the effects of sex, age, test year period, and test year nested within test year period; dogs were grouped into four levels based on age: 24, 25–29, 30–36 and 37–60 months, and **u** is the vector of the animal genetic effects. The prediction of **u** is called BLUP. It is also called the breeding value in animal and plant breeding; **e** is the vector of random residuals; and **X** and **Z** are incidence matrices associating **β** and **u** with **y**. It was assumed that 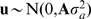 and 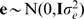, where **A** is the matrix of additive genetic relationships derived from identity by descent [24], [25], 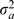 is the additive genetic variance, **I** is an identity matrix, and 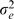 is the residual variance. Average information restricted maximum likelihood (AI-REML) method was used to estimate the variance components. The analysis was executed using the DMU Package [26], [27].

The accuracy of the breeding value was derived from Prediction Error Variance (PEV) and the additive (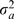) genetic variance by using the formula as follows:
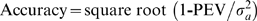



The maximum accuracy equals 1 and the minimum equals 0 [26], [27]. The inbreeding coefficient for each dog equals the diagonal element of the A matrix by subtracting one.

#### URLs

The breeding values, accuracy and inbreeding coefficient of the 258,851 Labrador retrievers can be accessed at the World Wide Web as follows: www.vet.cornell.edu/research/bvhip

## Supporting Information

Figure S1 User interface
(0.06 MB DOC)

Figure S2 Search results
(0.06 MB DOC)

## References

[1] Corley EA (1992). Role of the Orthopedic Foundation for Animals in the control of canine hip dysplasia.. Vet Clin North Am Small Anim Pract.

[2] Fluckiger MA, Friedrich GA, Binder H (1999). A radiographic stress technique for evaluation of coxofemoral joint laxity in dogs.. Vet Surg.

[3] Henricson B, Norberg I, Olsson SE (1966). On the etiology and pathogenesis of hip dysplasia: a comparative review.. J Small Anim Pract.

[4] Smith GK (1997). Advances in diagnosing canine hip dysplasia.. Journal of the American Veterinary Medical Association.

[5] Farese JP, Lust G, Williams AJ, Dykes NL, Todhunter RJ (1999). Comparison of measurements of dorsolateral subluxation of the femoral head and maximal passive laxity for evaluation of the coxofemoral joint in dogs.. American Journal of Veterinary Research.

[6] Farese JP, Todhunter RJ, Lust G, Williams AJ, Dykes NL (1998). Dorsolateral subluxation of hip joints in dogs measured in a weight-bearing position with radiography and computed tomography.. Veterinary Surgery.

[7] Henry GA (1992). Radiographic development of canine hip dysplasia.. Veterinary Clinics of North America: Small Animal Practice.

[8] Lust G, Todhunter RJ, Erb HN, Dykes NL, Williams AJ (2001). Repeatability of dorsolateral subluxation scores in dogs and correlation with macroscopic appearance of hip osteoarthritis.. American Journal of Veterinary Research.

[9] Swenson L, Audell L, Hedhammar A (1997). Prevalence and inheritance of and selection for hip dysplasia in seven breeds of dogs in Sweden and benefit: Cost analysis of a screening and control program.. Journal of the American Veterinary Medical Association.

[10] Leighton EA (1997). Genetics of canine hip dysplasia.. Journal of the American Veterinary Medical Association.

[11] Kaneene JB, Mostosky UV, Padgett GA (1997). Retrospective cohort study of changes in hip joint phenotype of dogs in the United States.. J Am Vet Med Assoc.

[12] Kaneene JB, Mostosky UV, Miller R (2009). Update of a retrospective cohort study of changes in hip joint phenotype of dogs evaluated by the OFA in the United States, 1989–2003.. Veterinary Surgery.

[13] Zhang Z, Zhu L, Sandler J, Friedenberg SS, Egelhoff J (2009). Estimation of heritabilities, genetic correlations, and breeding values of four traits that collectively define hip dysplasia in dogs.. American Journal of Veterinary Research.

[14] Van Vleck LD (1991). C. R. Henderson: farm boy, athlete, and scientist.. J Dairy Sci.

[15] Wood JL, Lakhani KH (2003). Effect of month of birth on hip dysplasia in labrador retrievers and Gordon setters.. Vet Rec.

[16] Wood JL, Lakhani KH, Dennis R (2000). Heritability of canine hip-dysplasia score and its components in Gordon setters.. Prev Vet Med.

[17] Wood JL, Lakhani KH, Dennis R (2000). Heritability and epidemiology of canine hip-dysplasia score in flat-coated retrievers and Newfoundlands in the United Kingdom.. Prev Vet Med.

[18] Janutta V, Hamann H, Distl O (2008). Genetic and phenotypic trends in canine hip dysplasia in the German population of German shepherd dogs.. Berl Munch Tierarztl Wochenschr.

[19] Ginja MM, Gonzalo-Orden JM, Melo-Pinto P, Bulas-Cruz J, Orden MA (2008). Early hip laxity examination in predicting moderate and severe hip dysplasia in Estrela mountain dog.. J Small Anim Pract.

[20] Henderson CR (1984). Applications of Linear Models in Animal Breeding.

[21] Zhu L, Zhang Z, Friedenberg S, Jung SW, Phavaphutanon J (2009). The long (and winding) road to gene discovery for canine hip dysplasia.. The Veterinary Journal.

[22] Gianola D, Heringstad B, Odegaard J (2006). On the quantitative genetics of mixture characters.. Genetics.

[23] Todhunter RJ, Bliss SP, Casella G, Wu R, Lust G (2003). Genetic structure of susceptibility traits for hip dysplasia and microsatellite informativeness of an outcrossed canine pedigree.. J Hered.

[24] Emik LO, Terrill CE (1949). Systematic procedures for calculating inbreeding coefficients.. J Hered.

[25] Cruden D (1949). The computation of inbreeding coefficients for closed populations.. J Hered.

[26] Madsen P, Jensen J (2007). A user's Guide to DMU.

[27] Madsen P, Sørensen P, Su G, Damgaard LH, Thomsen H (2006). DMU - a package for analyzing multivariate mixed models..

