# Supplementary material for: Retrospective Analysis for Genetic Improvement of Hip Joints of Cohort Labrador Retrievers in the United States: 1970–2007
Source: PLoS One. 2010 Feb 24;5(2):e9410. doi: 10.1371/journal.pone.0009410 (PMC2827553; doi:10.1371/journal.pone.0009410)
Supplement: Figure S1 — User interface (0.06 MB DOC) [file pone.0009410.s001.doc]

**Supplementary Information for:**

**Retrospective analysis for genetic improvement of hip scores of cohort Labrador Retrievers in the United States: 1970-2007**


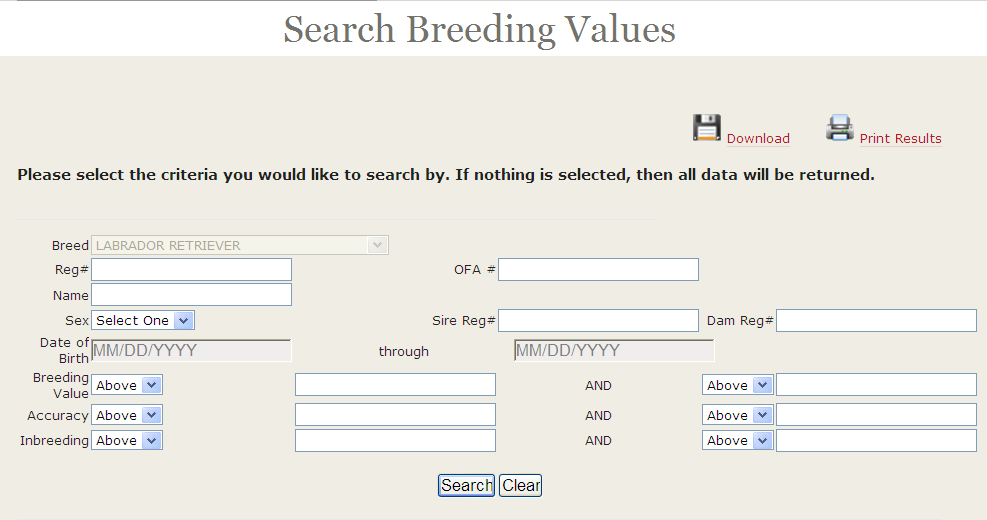


**Figure S1**. User interface for searching dogs through world-wide-web. The breeding values of Orthopedic Foundation for Animals (OFA) scores can be accessed publicly through world-wide-web ([www.vet.cornell.edu/research/bvhip](http://www.vet.cornell.edu/research/bvhip)). In addition to the search interface, the website also provides education materials, such as how to interpret breeding value and accuracy. Users can search dogs with a variety of criteria, such name, registration ID or OFA ID number, sex, and range of breeding values, accuracy and inbreeding coefficients. The search results can be displayed, printed or downloaded as Tab-delimitated text file.
