# Supplementary material for: Retrospective Analysis for Genetic Improvement of Hip Joints of Cohort Labrador Retrievers in the United States: 1970–2007
Source: PLoS One. 2010 Feb 24;5(2):e9410. doi: 10.1371/journal.pone.0009410 (PMC2827553; doi:10.1371/journal.pone.0009410)
Supplement: Figure S2 — Search results (0.06 MB DOC) [file pone.0009410.s002.doc]

**Supplementary Information for:**

**Retrospective analysis for genetic improvement of hip scores of cohort Labrador Retrievers in the United States: 1970-2007**


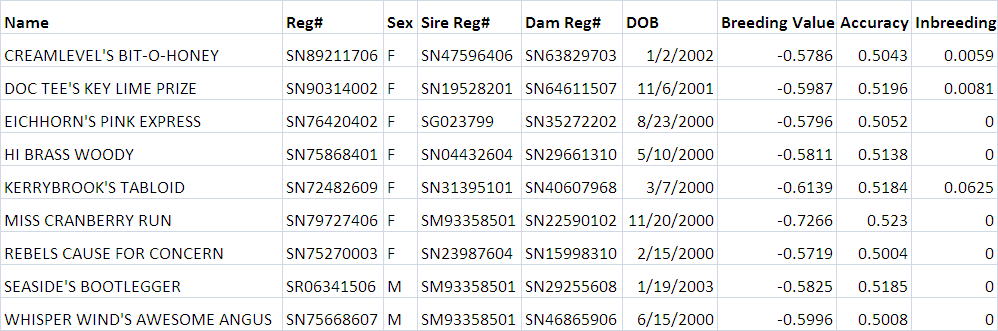


**Figure S2**. The top nine desirable dogs born after 2000. The dogs were selected by both breeding value and accuracy. As the category of excellent hip was coded as 1 and the worst hip category (severe) was code as 7, the lower breeding value, the better. A higher accuracy of breeding value requires many progeny tests. There are nine dogs with a breeding value below -0.57 and accuracy above 0.5. The search was performed on December 10, 2009 by using breeding value (<0.57) and accuracy (>0.5).
